# Intermittent Lighting Program Relieves the Deleterious Effect of Heat Stress on Growth, Stress Biomarkers, Physiological Status, and Immune Response of Broiler Chickens

**DOI:** 10.3390/ani12141834

**Published:** 2022-07-19

**Authors:** Abdulaziz A. Alaqil, Hanaa K. Abd El-Atty, Ahmed O. Abbas

**Affiliations:** 1 Department of Animal and Fish Production, College of Agricultural and Food Sciences, King Faisal University, P.O. Box 420, Al-Ahsa 31982, Saudi Arabia; 2 Department of Biotechnology Research, Animal Production Research Institute, Agricultural Research Center, Dokki, Giza 12611, Egypt; hanaa.amin@arc.sci.eg; 3 Department of Animal Production, Faculty of Agriculture, Cairo University, Gamma St., Giza 12613, Egypt

**Keywords:** heat stress, lighting program, stress indicators, physiological status, immune response, growth performance, broiler chickens

## Abstract

**Simple Summary:**

Chronic heat stress remains the most detrimental factor for broiler productivity in hot and desert regions. The manipulation of the lighting program is a useful and inexpensive tool to alleviate the negative effects of heat stress on broiler performance. The present study aimed to investigate the beneficial effects of an intermittent lighting (I.L.) program consisting of repeated periods of 1 h light to 3 h dark during a day on broiler performance under chronic heat-stress conditions. The results indicate that applying the I.L. program to heat-stressed broilers relieved the stress indicators and improved the immune response, physiological status, and growth performance of broilers. Therefore, the application of the I.L. program could be used as a beneficial strategy to recover broiler performance during heat-stress conditions.

**Abstract:**

The effects of heat stress on broiler performance and immunological response were explored using lighting-program manipulation as a potential tool. The study included 200 Cobb500 broiler chicks that were one day old at the time of recruitment. The birds were divided into four-compartment groups with similar environments (five cages per compartment, ten chicks per cage). Starting from the fourth day of age, birds of two compartments received a continuous lighting program (23L:1D a day; C.L. groups) while birds of the other two compartments received an intermittent lighting program (1L:3D 6 times per day; I.L. groups). Within each lighting program during 22–42 d of age, one group was subjected either to a thermoneutral temperature at 24 °C or heat stress at 35 °C. The results reveal that stress biomarkers, especially the plasma concentrations of corticosterone (CORT), tumor necrosis factor-alpha (TNF-α), and malondialdehyde (MDA) were relieved by 46%, 27%, and 51%, respectively, in the I.L. treatment groups compared to the C.L. program in broiler chicks subjected to heat stress. The liver function was also improved by 24% and 32% in AST and ALT levels, respectively, in the I.L. program compared to the C.L. program in stressed birds. Furthermore, the I.L. program positively influenced the immune response of the heat-stressed broilers. Eventually, the I.L. program increased the heat-stressed broilers’ body weight gain and feed conversion ratio. It can be concluded that applying the I.L. program to broiler chickens can effectively improve their physiological balance and growth performance under heat-stress conditions.

## 1. Introduction

High temperatures have long been known to be harmful to the poultry business. Chicken broilers are comparatively more susceptible to heat stress than other productive animals due to their lack of sweat glands, feather covering, and selection for high muscle mass [1]. Broilers under hot environmental conditions express a substantial reduction in productive performance such as feed intake, growth rate, and protein synthesis [2]. Heat stress has a deleterious impact on other elements of broiler production, including intestinal digestion, nutritional absorption, carcass features, immunological organ development, and survival [3].

Several physiological changes occur in broiler chickens subjected to long-term heat stress, which are detrimental to their well-being and productivity [4,5,6]. These deleterious effects may be attributed, in part, to the negative effects of heat stress on adaptive endocrine secretions such as insulin and thyroid hormones, which promote growth regulation, muscle metabolism, and protein synthesis [7]. Heat stress also affects broiler chickens’ immunological responses, alters serum metabolites, and causes redox imbalances [8,9,10]. Several studies have shown that heat stress negatively affects chickens’ immune systems, including a decrease in total white blood cells in circulation [11], a decrease in CD4+ and CD8+ cells in circulation [12], decreased antibody titers against sheep red blood cells [13], and decreased T- and B-lymphocyte proliferation [14]. It has been reported that heat stress induces spleen involution and disturbs the production of cytokines such as interleukin (IL)-4, IL-12, and interferon (IFN)-γ [15]. Additional immunosuppressive effects of heat stress were presented through different pathways in chickens such as the inhibition of innate immunity, up-regulation of apoptotic genes, high expression of cell death mediated proteins, depression of leukocyte protein synthesis, and increased levels of serum pro-inflammatory cytokines [16,17,18,19,20]. Heat stress in chickens also stimulates the hypothalamus–pituitary–adrenal axis, which results in the release of corticosterone (CORT) hormone [21]. Elevated CORT levels in heat-stressed broilers can disrupt metabolism, suppress the systemic immune response, and induce inflammation [22,23]. Furthermore, heat stress increases the production of reactive oxygen species (ROS), causing oxidative damage to chicken tissues and cells [24]. It was also revealed that oxidative stress and inflammation modulated by heat stress consequently impaired the mucosal and villus integrity of the small intestine as well as gut microbiome stability, which can be attributed to the compromised digestive and absorptive functions of broilers [25,26].

Heat stress in poultry can be alleviated by a multidisciplinary strategy that involves changing some surrounding factors, such as the ventilation system, bird density, and nutritional management [27]. The lighting system is also one of the environmental conditions that may positively affect broiler performance under normal [28] or heat-stress [29] conditions. Light is a primary tool used by birds to maintain biological functions and circadian rhythms of the body within a norm during the day [30]. Since near-continuous access to light can keep feed and water constantly visible to birds, which in turn maximizes feed consumption and growth, constant or near-constant lighting programs have been a traditional choice of photoperiod exposure in commercial broiler production [31]. However, other reports have found that the positive influence of photoperiod on feed intake and body weight gain of broilers continued to 21 d of age while a negative correlation of growth in broilers exposed to >12 h photoperiods was observed from 21–38 d of age [32]. It was found that the body weights and feed conversion ratio were better in broilers exposed to an intermittent lighting (I.L.) program of 1 h light (L): 3 h darkness (D) × 3 times per night than those in broilers exposed to a continuous lighting (C.L.) program of 24L:0D from 3–42 d of age [33]. It was also reported that the activity of splenic and peripheral T- and B-lymphocytes was higher in 6-week-old broilers grown on an I.L. program (1L:3D) than those grown on a C.L. (23L:1D) or intermediate lighting (12L:12D) program [34]. Moreover, broilers exposed to chronic heat stress exhibited a higher T-cell proliferation and cutaneous basophil hypersensitivity response accompanied by lower IL-1, IL-6, and tumor necrosis factor (TNF-α) proinflammatory cytokines and CORT levels when grown from 4–6 wk of age under an I.L. (1L:3D) program versus a C.L. (23L:1D) program [29]. Other studies reported that exposing broilers to different I.L. schedules of 18L:6D in total increased antioxidant status and decreased lipid peroxidation when compared to C.L. programs of 24L:0D [35]. Many reports indicated that C.L. programs could be more stressful to poultry than I.L. programs and can produce excessive free radicals that may harm the antioxidant status and immune system [36,37]. Furthermore, it was suggested that the beneficial effects of I.L. programs on broiler chickens might be due to the secretion of melatonin in the darkness (Kliger et al., 2000; Abbas et al., 2008; Zheng et al., 2013). Melatonin has a direct radical scavenging effect and plays a pivotal role in stimulating the antioxidant defense system [38]. In addition, melatonin reduces the heterophil to lymphocyte (H/L) ratio and has a stimulatory effect on lymphocyte proliferation, antibody formation, and IL-2, IL-6, and INF-γ cytokine secretion [34,35,39,40].

Various information is available on various solutions for mitigating the negative consequences of extreme heat in broiler chickens. However, little is known about the impact of the lighting-schedule program on broiler productivity and physiological performance, particularly under heat stress. As a result, the goal of this study was to see how effective intermittent light programs are in reversing the growth, physiological, and immunological damage caused by chronic heat stress in broiler chickens.

## 2. Materials and Methods

### 2.1. Ethics Compliance Statement

The ethics council of Saudi Arabia’s King Faisal University authorized the study’s experimental protocols (KFU-REC-2022-MAR-EA000533). It is dedicated to minimizing avian suffering and reducing discomfort, agony, and sorrow caused by heat stress. Birds with any evidence of chronic stress, such as hard breathing, releasing fluids from the beak, loss of appetite, crimped feathers, or a drooping appearance, were quickly euthanized through cervical dislocation. 

### 2.2. Experimental Design

A local hatchery provided us with 200 one-day-old male broiler chicks “Cobb500™,” which we randomly placed in broiler battery cages in four identical chambers for the duration of the study (5 cages per chamber, 10 chicks per cage). These cages, which come with wire floors of 1.5 mm thickness, measured 125 cm length × 90 cm width × 60 cm height. The chambers were environmentally controlled and measured an area of 30 m^2^ each (10 m × 3 m). According to the Cobb-500 broiler management plan [41], a base diet of corn–soybean meal was developed. Table 1 shows the basic diet elements and nutritional makeup. During the experiment, birds were given unlimited access to feed and water. All birds were kept under 24 h lighting for the first three days of their lives. From 4 to 42 days of age, birds in the first and second chambers received a constant lighting program of 23 h light and 1 h dark daily (C.L. treatment groups), while the third and fourth chambers received an intermittent lighting program of 1 h light and 3 h dark repeated 6 times per day (I.L. treatment groups). For the first three days, all chambers were kept at 33 °C, then decreased to 30 °C for the next four days. All chambers were then cooled by 2 °C every week until the chicks were 21 days old. From 22–42 d of age, birds in the first and third chambers were exposed to a thermoneutral temperature at 24 °C. Birds in the heat-stress groups (the second and fourth chambers) were exposed to heatwave conditions of 35 °C for 8 h per day (from 9:00 to 17:00), with the temperature reduced to 24 °C outside of these times. The four compartments had their relative humidity set at 50%. Growth performance data were obtained during the period of 22–42 d of age, considering the chambers as the experimental groups and each replicate cage in the chamber as the experimental unit. For the purposes of studying stress indicators, blood metabolites, and immunological response, blood samples were taken from birds at 42 days old. 

### 2.3. Growth Performance

Body weight was individually measured at 22 (initial body weight, IBW) and 42 days of age (final body weight, FBW). For each experimental group, the body weight gain (BWG) was determined per bird for the corresponding period (22–42 d of age). Feed intake (FI) was calculated for each cage by taking the leftover feed from the total amount of given feed. The feed conversion ratio (FCR) was then calculated by dividing the amount of feed each animal ate by the amount of weight it gained. This was calculated for each cage (replicate) in each experimental group (chamber).

### 2.4. Stress Biomarkers

As soon as the treatments were over, two blood samples from the brachial vein were taken and inserted into heparinized tubes for each replicate per experimental group (*n* = 10). The birds were quickly sampled within 3 min during the night to avoid affecting stress indicators by handling [43]. A volume of 10 µL blood was dropped to measure the heterophil/lymphocyte (H/L) ratio as stress indicator. Briefly, the blood drop was smeared on a glass slide, then fixed with methanol. They were then stained using Hema-3 solutions (Fisher Scientific, Pittsburg, PA, USA). Up to 200 white blood cells were studied under a microscope with oil immersion at 1000× magnification. The H/L ratio was then discovered [44].

The blood sample was then centrifuged for 10 min at 2000× *g* at 4 °C to separate the plasma, which can then be used to figure out other stress biomarkers, such as corticosterone (CORT), tumor necrosis factor alpha (TNF-α), and malondialdehyde (MDA). In brief, plasma CORT and TNF-α were assayed according to the manufacturer protocol of chicken ELISA kits (MBS701668, MBS2509660, respectively) (MyBioSource, Inc., San Diego, CA, USA). The principal of assay is based on mixing the sample with a specific antibody in pre-coated microplates then supplementing a conjugated reagent. After washing, a color was raised by adding an appropriate ELISA substrate to finally measure the optical density at 450 nm by using a microplate reader (ELx808™, BioTek Instruments, Winooski, VT, USA). Plasma MDA assay was conducted using a colorimetric test kit (MBS9718963, MyBioSource, Inc., San Diego, CA, USA) to determine the lipid peroxidation product. The absorbance was measured at 532 nm using a microplate reader. The complete assay steps are described in the Supplementary Materials File S1: Stress biomarkers assay.

### 2.5. Blood Metabolites

Two blood samples per replication in each experimental group (*n* = 10) were taken at the conclusion of the treatments (42 d of age) and the plasma was separated by centrifuging the samples at 2000× *g* for 10 min at 4 °C. The plasma was then be used to measure some metabolites, including the total protein (TP), aspartate aminotransferase (AST), alanine aminotransferase (ALT), and chicken triiodothyronine (chT_3_). Briefly, plasma TP was detected using the Bradford assay kit (ab102535, Abcam, Waltham, MA, USA), which produces a blue complex detected at 595 nm. For the plasma AST and ALT, available colorimetric kits (ab241035 and ab105135, respectively) were used from Abcam. The reaction mix was added to the samples and allowed for incubation at 37 °C. The optical density at 450 nm for AST or 570 nm for ALT was obtained after 10 and 60 min to determine the amount of glutamate or pyruvate generated per min by AST or ALT, respectively. The concentration of T_3_ in the plasma was measured according to the guidelines of ELISA kits specific for chickens (MBS269454; MyBioSource, Inc.). The kit’s reagents included biotinylated chT3 antibody, enzyme-conjugate, and color reagents A, B and C. After processing the protocol steps, the standard solution color gradients developed, and the optical density was then measured at 450 nm. The complete assay steps for blood metabolites are detailed in the Supplementary Materials File S1. 

### 2.6. Immune Response

At the end of the 42-day experiment, blood samples were taken from broiler birds (*n* = 10; 2 samples were taken from each replicate in each treatment group) and put into heparinized tubes. For measurement of total white blood cells (TWBC), a mixture drop of the entire sample diluted with brilliant cresyl blue stain solution was put on a hemocytometer slide and examined with a microscope at a magnification of 200× to look at the TWBC and count them [45]. The remaining blood samples (*n* = 10) were used to measure the stimulation index (SI) of T- and B-lymphocyte proliferation, as reported in a recent study [46]. First, the peripheral blood mononuclear cells (PBMC) were separated by a separation medium. The concentration of viable lymphocytes in each sample was detected by Trypan Blue dye and then re-adjusted at 10 million cells/mL in triplicates in 96-well plates. The T- or B-lymphocyte proliferation were promoted with Concanavalin-A mitogen or Lipopolysaccharide, respectively. The cells were then incubated with 3-[4,5-dimethylthiazol]-2,5-diphenyltetrazolium bromide (MTT) followed by addition of sodium dodecyl sulfates. Finally, the optical density at 570 nm (OD570) was measured for stimulated to unstimulated cells in each sample to compute the SI of T- and B-lymphocytes. More details on the immune response parameters assay are provided in the Supplementary Materials File S1. 

The broiler antibody titers against sheep red blood cells (Anti-SRBCs AB) were determined using procedures outlined in a prior study [47]. In short, one week before the end of the experiment, two birds per replication in each treatment group (*n* = 10) were injected with 1 mL of 5 percent SRBC. Blood samples were taken from the birds at 42 days of age, and the sera were separated. Serial doubling dilutions of sera were pipetted with 2% SRBC solution then were left overnight. The last dilution with positive agglutination was used to explore the antibody titer values as indicated in the Supplementary File S1. 

### 2.7. Statistical Analysis

Data were assessed for homogeneity using Levene’s test. A two-way ANOVA using a general linear model was used to analyse the data (SPSS 2013, Software Package version 22.0, I.B.M. corp., Armonk, NY, USA). The main effects were the heat stress (24 °C vs. 35 °C), the light program (C.L. vs. I.L.), and their interaction. The results of interactions were displayed as mean values ± standard error of means (SEM), and the *p*-values of main effects were shown. Mean differences were tested at a 0.05 level of significance using the post hoc Duncan’s test. 

## 3. Results

### 3.1. Growth Performance

Heat stress cyclic waves had a deleterious impact on all elements of broiler development that were evaluated. Table 2 shows the results of broiler growth performance as impacted by heat stress, light program, and their interaction. Compared to the broilers in the thermoneutral group, the FBW, BWG, and FI were substantially (*p* < 0.05) reduced by 20, 29, and 16 percent, respectively, in the broilers exposed to heat stress. Broilers that were exposed to heat stress had greater FCR values (*p* < 0.05), indicating lower production. Compared to the birds that underwent the C.L. program, the birds who received the I.L. program exhibited a substantial rise in BWG and better FCR. Moreover, birds that received the I.L. program under heat-stress conditions showed better (*p* < 0.05) performance in the FBW by 13% and in the BWG by 20%, with a considerable increase in the FCR compared with birds that received the C.L. program in the heat-stressed group.

### 3.2. Stress Biomarkers

As demonstrated in Table 3, heat stress, light program, and their combination had a significant influence (*p* < 0.05) on the stress biomarkers studied. It was found that the amount of heat stress substantially increased the H/L ratio (0.79 vs. 0.39), CORT (6.49 vs. 2.00 ng/mL), TNF-α (166.95 vs. 98.39 pg/mL), and MDA (3.89 vs. 1.10 µmol/mL) levels compared to the thermoneutral condition. In contrast, all stress biomarkers were significantly decreased under the I.L. program vs. the C.L. program (0.52 vs. 0.67 H/L ratios, 3.22 vs. 5.28 ng/mL CORT, 117.62 vs. 147.73 pg/mL TNF-α, and 1.82 vs. 3.17 µmol/mL MDA, respectively). Furthermore, under heat-stress conditions, the I.L. program ameliorated the elevation in the H/L ratio, CORT, TNF-α, and MDA levels by 29, 46, 27, and 51%, respectively, in comparison with the C.L. program. 

### 3.3. Blood Metabolites

The results of the blood metabolites assay as influenced by heat stress, light program, and their interaction are displayed in Table 4. Heat stress raised plasma concentrations considerably (*p* < 0.05). The TP, AST, and ALT increased by 46, 65, and 95%, respectively, while the chT_3_ decreased by 43%, compared to the thermoneutral condition. In contrast, birds who received the I.L. program expressed lower levels of TP, AST, and ALT and higher levels of chT3 compared to those levels in the birds who received the C.L. program. In addition, the I.L. treatment significantly (*p* < 0.05) alleviated the negative effect of heat stress on TP, AST, ALT, and chT_3_ by 26, 24, 32, and 88%, respectively, compared to the C.L. treatment in the heat-stressed broilers.

### 3.4. Immune Response

The effects of heat stress, light program, and their interaction on the immune response of broilers are shown in Table 5. Results indicate that heat stress (*p* < 0.05) inhibited the immune response of broilers when compared to broilers in the thermoneutral group (34.03 vs. 55.34 × 10^3^ TWBC/mL, 3.32 vs. 6.29 T-lymphocyte SI, 1.62 vs. 2.84 B-lymphocyte SI, and 3.93 vs. 6.95 anti-SRBC AB titer for heat-stressed birds vs. thermoneutral birds, respectively). Birds exposed to heat stress under the I.L. program exhibited significantly (*p* < 0.05) higher TWBC, T- and B-lymphocyte SI, and anti-SRBC-AB titers than birds exposed to heat stress under the C.L. program. However, these values were still significantly lower than those in birds of the thermoneutral group under either C.L. or I.L. programs. Furthermore, birds that received the I.L. program in the thermoneutral condition showed higher (*p* < 0.05) TWBC, T- and B-lymphocytes SI than those birds that received the C.L. program in the thermoneutral condition (Table 5). 

## 4. Discussion

The negative impact of high environmental temperatures on commercial broiler production has been well documented. When broiler chickens were exposed to cyclic heat stress from 22–42 d of age in the current study, their growth, physiological and immunological performance were negatively affected. In agreement with the results of the current study, the reduction in TWBC, lymphocyte proliferation, and antibody production in broilers by exposure to chronic or cyclic heat stress was earlier demonstrated in previous works [19,48,49,50,51]. Heat stress also resulted in a significant increase in CORT levels, which is secreted by the activation of the hypothalamic–pituitary–adrenal (HPA) axis after exposure to chronic heat stress [52]. The results of the present study and previous studies displayed that the high levels of CORT in the heat-stressed birds could be the key factor that induces the elevation in the other stress biomarkers such as the H/L ratio, TNF-α, and MDA [53,54], and the physiological deterioration such as the increasing TP, AST and ALT, and decreasing chT_3_ levels [20], as well as the depression of the immune response [55]. Such negative effects of heat stress on immune function are distinctly reflected in the growth performance of broilers. As represented in Table 2, FI was significantly decreased in HS-broilers, and this, in part, may limit the metabolic energy needed for protein synthesis and subsequent growth [56]. In addition, it was suggested that the low performance of HS-broilers could be correlated with the inhibition of the elongation phase of the protein translation process, which, in turn, restrains the metabolic pathway and protein synthesis of body tissues [19].

In the C.L. programs, the broilers are subjected to a constant photoperiod of 23–24 h daily to provide more access time to feeders and, subsequently, maximize the feed intake and body weight gain [57]. The I.L. programs, repeating cycles of light and darkness in a 24 h period, have been suggested as alternative lighting programs to contribute to broiler chickens’ growth and economic performance without compromising other physiological and immune functions [35]. When reared under thermoneutral conditions in the present study, broilers exposed to I.L and C.L. programs did not exhibit significant differences in the growth performance traits, major stress biomarkers, and blood metabolites. However, the C.L. programs were found to be a stressful environmental factor to the birds and may be a reason for excess free radical generation in the cells [36,37]. This may explain the increase in TNF-α when applying C.L. programs in the thermoneutral broilers. Decreases in TWBC, as well as T- and B-lymphocytes, were also observed in these broilers, indicating that birds exposed to such continuous lighting programs may become more immunologically fragile [57]. 

The results of the present study illustrate a critical (*p* < 0.05) interaction impact of heat stress and lighting program on all aspects of broiler performance. The maximum levels of stress biomarkers were recorded when using the C.L. program under heat-stress conditions, while adopting the I.L. program with birds under heat stress ameliorated the levels of stress biomarkers (Table 3). A possible reason for this may be the secretion of melatonin during darkness in the I.L. program, which directly or indirectly activates antioxidant enzymes [35] and decreases stress biomarkers such as MDA [58]. It was also reported that melatonin has an inhibitory effect on the expression of nuclear factor-kappa β (NF-κβ), which regulates the transcription of pro-inflammatory cytokines such as TNF-α [59]. In addition, the reduction in MDA levels in the I.L. program could be attributed to the reduction in endogenous heat production as a result to the low metabolic rates during darkness [60]. The reduction in TNF-α in the heat-stressed birds that received the I.L. program compared to those that received C.L. could be attributed to the decrease in the CORT levels in the same group, as this correlation between CORT secretion and TNF-α expression has been previously reported in laying hens [61]. In addition, CORT reduction in the I.L. heat-stressed group directly caused a substantial decrease in the H/L ratio through the responsivity of leukocyte distribution to the physiological regulating actions of glucocorticoids [62]. 

In the present study, a significant elevation was observed in the immune response of birds in the I.L. program compared to birds in the C.L. program under heat-stress conditions. The stimulation of lymphocyte proliferation in the I.L. heat-stress group may be due to the decrease in CORT and the subsequent increase in the lymphocyte numbers [63]. In contrast, the secretion of melatonin in the birds that received the I.L. program can stimulate lymphocyte proliferation under heat-stress conditions by enhancing the secretion of immune mediators such as cytokines [64]. The I.L. program also enhanced the TWBC and antibody production against SRBC under heat-stress conditions due to the direct effect of melatonin secretion or the indirect effect of other endocrine hormones such as thyroid hormone [29]. 

The healthy liver orchestrates the metabolism of proteins and amino acids [65]. On the other hand, the maximum TP, AST, and ALT levels were observed in the heat-stressed birds with the C.L. program, indicating the harmful effect of both stress factors (high temperature and continuous light) on the liver functions. In contrast, the lowest levels of chT3 were obtained in the same stressed group. These negative effects of stress on physiological parameters have been previously explained by the direct effect of CORT on protein catabolism [66], liver impairment [67], and peripheral deiodinase inhibition [68]. The physiological status of heat-stressed broilers was improved when they received the I.L. program (Table 4). The results indicated that the I.L. program could ameliorate the deleterious effects of heat stress on the liver tissues of broilers. Moreover, melatonin secretion during the darkness of the I.L. program can elevate the plasma leptin and, consequently, the plasma T_3_ and T_4_ hormones [69]. In agreement with a previous study [37], the lighting program did not significantly affect the F.I. of broilers in the current experiment. However, the FBW, BWG, and FCR were better in the broilers that received the I.L. program than those that received the C.L. program under heat-stress conditions (Table 2). This result could be correlated with the lower physical activity and energy expenditure of those chickens during the dark periods in the I.L. program [70]. These findings show that applying an I.L. program with heat-stressed broilers helps them to recover their normal physiological status and growth performance. 

## 5. Conclusions

The present study provides new information on the beneficial application of the I.L. programs of 1L:3D cycles for broiler chickens during heat-stress conditions. The heat-stressed birds displayed lower stress biomarkers when receiving I.L. than in the C.L. program. In addition, the immunosuppression signs induced by heat stress were alleviated by using the I.L. program. In addition, liver function and physiological status were maintained when the birds received the I.L. program under heat-stress conditions. These favorable impacts of the I.L. program eventually trickled down to broiler chicks kept under prolonged heat stress. Considering these data, it is possible to infer that using the I.L. program to recover broiler performance during heat-stress circumstances is a viable option. 

## Figures and Tables

**Table 1:** Ingredients and nutritional composition of the basal diet.

Ingredients (g/kg)	Starter (0–8 d)	Grower (9–28 d)	Finisher (29–42 d)
Corn	607.0	654.0	693.0
Gluten meal	70.0	50.0	50.0
Soybean meal, 48% CP	289.0	243.0	203.0
Soybean oil	0.0	20.0	22.0
Di-calcium phosphate	4.0	4.0	4.0
Limestone	20.0	19.0	18.0
salt	4.5	4.5	4.5
Vitamin-Mineral Premix ^1^	5.5	5.5	5.5
Nutritional composition			
Dry matter (g/kg) ^2^	906.0	901.0	908.9
Total ash (g/kg) ^2^	55.0	53.0	39.1
Crude protein (g/kg) ^2^	229.8	199.8	184.6
Crude fat (g/kg) ^2^	58.3	77.5	83.4
Crude fiber (g/kg) ^2^	32.0	35.0	35.8
Metabolizable energy (MJ/kg) ^3^	12.6	13.1	13.3
L-lysine (g/kg) ^3^	12.1	11.6	10.4
DL-Methionine (g/kg) ^3^	4.8	4.7	4.3
Calcium (g/kg) ^3^	9.1	8.6	8.1
Available phosphorus (g/kg) ^3^	4.5	4.2	4.1

^1^ Premix provide the following components per kg of the basal diet: vitamins A 10 KIU, D_3_ 5 KIU, E 65 IU, K 3 mg, B_1_ 3 mg, B_2_ 9 mg, B_6_ 4 mg, B_12_ 0.02 mg, biotin 0.20 mg, niacin 20 mg, pantothenic acid 15 mg, folic acid 2 mg, and choline chloride 500 mg; and minerals Mn 100 mg, Fe 40 mg, Zn 100 mg, Cu 15 mg, Se 0.35 mg, and Iodine 1 mg. ^2^ Determined values [42]. ^3^ Calculated values.

**Table 2:** Effect of heat stress (HS), light program (LP), and their interaction (HS∗LP) on growth performance of broiler chicks.

Parameters	Thermoneutral		Heat Stress		SEM (*n*)	*p*-Value		
	C.L.	I.L.	C.L.	I.L.		HS	LP	HS∗LP
IBW (g)	750	736	731	747	33.3 (50)	NS	NS	NS
FBW (g)	2341 ^a^	2410 ^a^	1780 ^c^	2010 ^b^	93.7 (50)	0.016	NS	0.044
BWG (g)	1591 ^a^	1674 ^a^	1049 ^c^	1263 ^b^	82.5 (50)	<0.001	0.013	0.039
FI (g)	2955 ^a^	3025 ^a^	2523 ^b^	2497 ^b^	147.3 (50)	0.007	NS	NS
FCR	1.86 ^c^	1.81 ^c^	2.41 ^a^	1.98 ^b^	0.033 (50)	0.024	0.047	0.031

^a,b,c^ Means in the same row with uncommon superscripts are significantly different (*p* < 0.05). Abbreviations: IBW, initial body weight at 22 d of age; FBW, final body weight at 42 d of age; BWG, body weight gain; FI, feed intake; FCR, feed conversion ratio; C.L., continuous light (23L:1D); I.L., intermittent light (1L:3D); SEM (*n*), standard error of means (number of observations); NS, non-significant (*p* > 0.05).

**Table 3:** Effect of heat stress (HS), light program (LP) and their interaction (HS∗LP) on stress biomarkers of broiler chicks.

Parameters	Thermoneutral		Heat Stress			*p*-Value		
	C.L.	I.L.	C.L.	I.L.	SEM (*n*)	HS	LP	HS∗LP
H/L ratio	0.41 ^c^	0.37 ^c^	0.93 ^a^	0.66 ^b^	0.07 (10)	<0.001	0.006	0.047
CORT, ng/mL	2.14 ^c^	1.87 ^c^	8.41 ^a^	4.57 ^b^	0.67 (10)	<0.001	0.002	0.002
TNF-α, pg/mL	102.91 ^c^	93.87 ^d^	192.55 ^a^	141.36 ^b^	6.71 (10)	<0.001	<0.001	0.002
MDA, µmol/mL	1.13 ^c^	1.07 ^c^	5.21 ^a^	2.57 ^b^	0.31 (10)	<0.001	0.007	0.004

^a,b,c,d^ Means in the same row with uncommon superscripts are significantly different (*p* < 0.05). Abbreviations: CORT, corticosterone; TNF-α, tumor necrosis factor-alpha; MDA, malondialdehyde; H/L ratio, heterophils to lymphocytes ratio; C.L., continuous light (23L:1D); I.L., intermittent light (1L:3D); SEM (*n*), standard error of means (number of observations).

**Table 4:** Effect of heat stress (HS), light program (LP), and their interaction (HS∗LP) on blood metabolites of broiler chicks.

Parameters	Thermoneutral		Heat Stress			*p*-Value		
	C.L.	I.L.	C.L.	I.L.	SEM (*n*)	HS	LP	HS∗LP
TP, g/dL	3.46 ^c^	3.54 ^c^	5.88 ^a^	4.36 ^b^	0.28 (10)	<0.001	0.001	0.043
AST, U/mL	86.49 ^c^	81.34 ^c^	157.34 ^a^	118.97 ^b^	4.77 (10)	<0.001	0.013	0.025
ALT, U/mL	11.14 ^c^	10.87 ^c^	25.61 ^a^	17.36 ^b^	1.29 (10)	<0.001	0.021	0.033
chT_3_, µmol/ml	5.23 ^a^	5.88 ^a^	2.19 ^c^	4.12 ^b^	0.39 (10)	<0.001	0.005	0.037

^a,b,c^ Means in the same row with uncommon superscripts are significantly different (*p* < 0.05). Abbreviations: TP, total protein; T_3_, triiodothyronine; AST, aspartate aminotransferase; ALT, alanine aminotransferase; C.L., continuous light (23L:1D); I.L., intermittent light (1L:3D); SEM (*n*), standard error of means (number of observations).

**Table 5:** Effect of heat stress (HS), light program (LP), and their interaction (HS∗LP) on immune response of broiler chicks.

Parameters	Thermoneutral		Heat Stress			*p*-Value		
	C.L.	I.L.	C.L.	I.L.	SEM (*n*)	HS	LP	HS∗LP
TWBC, 10^3^/mL	49.41 ^b^	61.26 ^a^	29.63 ^d^	38.43 ^c^	3.48 (10)	<0.001	<0.001	0.019
T-lymphocytes SI	5.36 ^b^	7.21 ^a^	2.49 ^d^	4.15 ^c^	0.47 (10)	<0.001	0.003	0.029
B-lymphocytes SI	2.51 ^b^	3.17 ^a^	0.91 ^c^	2.33 ^b^	0.24 (10)	<0.001	0.001	0.008
Anti-SRBCs AB, log_2_	6.78 ^a^	7.11 ^a^	3.19 ^c^	4.66 ^b^	0.63 (10)	0.002	0.014	0.036

^a,b,c,d^ Means in the same row with uncommon superscripts are significantly different (*p* < 0.05). Abbreviations: TWBC, total white blood cells; SI, stimulation index; Anti-SRBCs AB, anti-sheep red blood cells antibody; C.L., continuous light (23L:1D); I.L., intermittent light (1L:3D); SEM (*n*), standard error of means (number of observations).

## Data Availability

Not applicable.

## Supplementary Materials

The following supporting information can be downloaded at: https://www.mdpi.com/article/10.3390/ani12141834/s1, File S1: stress biomarkers assay, blood metabolites assay, and immune response parameters assay.

## References

[1] Renaudeau D., Collin A., Yahav S., de Basilio V., Gourdine J.L., Collier R.J. (2012). Adaptation to hot climate and strategies to alleviate heat stress in livestock production. Animal.

[2] Temim S., Chagneau A.M., Peresson R., Tesseraud S. (2000). Chronic Heat Exposure Alters Protein Turnover of Three Different Skeletal Muscles in Finishing Broiler Chickens Fed 20 or 25% Protein Diets. J. Nutr..

[3] Gonzalez-Rivas P.A., Chauhan S.S., Ha M., Fegan N., Dunshea F.R., Warner R.D. (2020). Effects of heat stress on animal physiology, metabolism, and meat quality: A review. Meat Sci..

[4] Lara L.J., Rostagno M.H. (2013). Impact of heat stress on poultry production. Animals.

[5] Liu L., Ren M., Ren K., Jin Y., Yan M. (2020). Heat stress impacts on broiler performance: A systematic review and meta-analysis. Poult. Sci..

[6] Awad E.A., Najaa M., Zulaikha Z.A., Zulkifli I., Soleimani A.F. (2019). Effects of heat stress on growth performance, selected physiological and immunological parameters, caecal microflora, and meat quality in two broiler strains. Asian-Australas. J. Anim. Sci..

[7] Boussaid-Om Ezzine S., Everaert N., Métayer-Coustard S., Rideau N., Berri C., Joubert R., Temim S., Collin A., Tesseraud S. (2010). Effects of heat exposure on Akt/S6K1 signaling and expression of genes related to protein and energy metabolism in chicken (Gallus gallus) pectoralis major muscle. Comp. Biochem. Physiol. Part B Biochem. Mol. Biol..

[8] He S., Yin Q., Xiong Y., Li J., Liu D. (2020). Characterization of heat stress affecting the growth performance, blood biochemical profile, and redox status in male and female broilers at market age. Trop. Anim. Health Prod..

[9] Habibu B., Dzenda T., Ayo J.O., Yaqub L.S., Kawu M.U. (2018). Haematological changes and plasma fluid dynamics in livestock during thermal stress, and response to mitigative measures. Livest. Sci..

[10] Niu Z.Y., Liu F.Z., Yan Q.L., Li W.C. (2009). Effects of different levels of vitamin E on growth performance and immune responses of broilers under heat stress. Poult. Sci..

[11] Mashaly M.M., Hendricks G.L., Kalama M.A., Gehad A.E., Abbas A.O., Patterson P.H. (2004). Effect of Heat Stress on Production Parameters and Immune Responses of Commercial Laying Hens. Poult. Sci..

[12] Xu Y., Lai X., Li Z., Zhang X., Luo Q. (2018). Effect of chronic heat stress on some physiological and immunological parameters in different breed of broilers. Poult. Sci..

[13] Olfati A., Mojtahedin A., Sadeghi T., Akbari M., Martínez-Pastor F. (2018). Comparison of growth performance and immune responses of broiler chicks reared under heat stress, cold stress and thermoneutral conditions. Span. J. Agric. Res..

[14] Hirakawa R., Nurjanah S., Furukawa K., Murai A., Kikusato M., Nochi T., Toyomizu M. (2020). Heat Stress Causes Immune Abnormalities via Massive Damage to Effect Proliferation and Differentiation of Lymphocytes in Broiler Chickens. Front. Vet. Sci..

[15] Ohtsu H., Yamazaki M., Abe H., Murakami H., Toyomizu M. (2015). Heat Stress Modulates Cytokine Gene Expression in the Spleen of Broiler Chickens. J. Poult. Sci..

[16] Ma D., Liu Q., Zhang M., Feng J., Li X., Zhou Y., Wang X. (2019). iTRAQ-based quantitative proteomics analysis of the spleen reveals innate immunity and cell death pathways associated with heat stress in broilers (*Gallus gallus*). J. Proteom..

[17] Abbas A.O., Alaqil A.A., El-Beltagi H.S., Abd El-Atty H.K., Kamel N.N. (2020). Modulating laying hens productivity and immune performance in response to oxidative stress induced by e. Coli challenge using dietary propolis supplementation. Antioxidants.

[18] Alzarah M.I., Althobiati F., Abbas A.O., Mehaisen G.M.K., Kamel N.N. (2021). Citrullus colocynthis seeds: A potential natural immune modulator source for broiler reared under chronic heat stress. Animals.

[19] Kamel N.N., Ahmed A.M.H., Mehaisen G.M.K., Mashaly M.M., Abass A.O. (2017). Depression of leukocyte protein synthesis, immune function and growth performance induced by high environmental temperature in broiler chickens. Int. J. Biometeorol..

[20] Mehaisen G.M.K.K., Eshak M.G., Elkaiaty A.M., Atta A.R.M., Mashaly M.M., Abass A.O. (2017). Comprehensive growth performance, immune function, plasma biochemistry, gene expressions and cell death morphology responses to a daily corticosterone injection course in broiler chickens. PLoS ONE.

[21] Padgett D.A., Glaser R. (2003). How stress influences the immune response. Trends Immunol..

[22] Quinteiro-Filho W.M., Ribeiro A., Ferraz-de-Paula V., Pinheiro M.L., Sakai M., Sá L.R.M., Ferreira A.J.P., Palermo-Neto J. (2010). Heat stress impairs performance parameters, induces intestinal injury, and decreases macrophage activity in broiler chickens. Poult. Sci..

[23] Zaytsoff S.J.M., Brown C.L.J., Montina T., Metz G.A.S., Abbott D.W., Uwiera R.R.E., Inglis G.D. (2019). Corticosterone-mediated physiological stress modulates hepatic lipid metabolism, metabolite profiles, and systemic responses in chickens. Sci. Rep..

[24] Hu R., He Y., Arowolo M.A., Wu S., He J. (2019). Polyphenols as Potential Attenuators of Heat Stress in Poultry Production. Antioxidants.

[25] Santos R.R., Awati A., Roubos-van den Hil P.J., van Kempen T.A.T.G., Tersteeg-Zijderveld M.H.G., Koolmees P.A., Smits C., Fink-Gremmels J. (2019). Effects of a feed additive blend on broilers challenged with heat stress. Avian Pathol..

[26] Shi D., Bai L., Qu Q., Zhou S., Yang M., Guo S., Li Q., Liu C. (2019). Impact of gut microbiota structure in heat-stressed broilers. Poult. Sci..

[27] Dayyani N., Bakhtiari H. (2013). Heat stress in poultry: Background and affective factors. Int. J. Adv. Biol. Biomed. Res..

[28] Yang H., Xing H., Wang Z., Xia J., Wan Y., Hou B., Zhang J. (2016). Effects of Intermittent Lighting on Broiler Growth Performance, Slaughter Performance, Serum Biochemical Parameters and Tibia Parameters. Ital. J. Anim. Sci..

[29] Abbas A.O., Gehad A.E., Hendricks G.L., Gharib H.B.A., Mashaly M.M. (2007). The effect of lighting program and melatonin on the alleviation of the negative impact of heat stress on the immune response in broiler chickens. Int. J. Poult. Sci..

[30] Rattenborg N.C., Obermeyer W.H., Vacha E., Benca R.M. (2005). Acute effects of light and darkness on sleep in the pigeon (*Columba livia*). Physiol. Behav..

[31] Ingram D.R., Hatten L.F., McPherson B.N. (2000). Effects of Light Restriction on Broiler Performance and Specific Body Structure Measurements. J. Appl. Poult. Res..

[32] Lien R.J., Hess J.B., McKee S.R., Bilgili S.F., Townsend J.C. (2007). Effect of light intensity and photoperiod on live performance, heterophil-to-lymphocyte ratio, and processing yields of broilers. Poult. Sci..

[33] Petek M., Sönmez G., Yildiz H., Baspinar H. (2005). Effects of different management factors on broiler performance and incidence of tibial dyschondroplasia. Br. Poult. Sci..

[34] Kliger C.A., Gehad A.E., Hulet R.M., Roush W.B., Lillehoj H.S., Mashaly M.M. (2000). Effects of photoperiod and melatonin on lymphocyte activities in male broiler chickens. Poult. Sci..

[35] Zheng L., Ma Y.E., Gu L.Y., Yuan D., Shi M.L., Guo X.Y., Zhan X.A. (2013). Growth performance, antioxidant status, and nonspecific immunity in broilers under different lighting regimens. J. Appl. Poult. Res..

[36] EI-Badry A., Abdel-Fattah S., Moslim G. (2015). Effect of Early Heat Conditioning and Lighting Regime on Physiological and Immune Responses of Muscovy Ducks During Summer Season. J. Anim. Poult. Prod..

[37] Abbas A.O., El-Dein A.K.A., Desoky A.A., Galal M.A.A. (2008). The effects of photoperiod programs on broiler chicken performance and immune response. Int. J. Poult. Sci..

[38] Tomás-Zapico C., Coto-Montes A. (2005). A proposed mechanism to explain the stimulatory effect of melatonin on antioxidative enzymes. J. Pineal Res..

[39] Brennan C.P., Hendricks G.L., El-Sheikh T.M., Mashaly M.M. (2002). Melatonin and the Enhancement of Immune Responses in Immature Male Chickens. Poult. Sci..

[40] Haldar C., Ahmad R. (2010). Photoimmunomodulation and melatonin. J. Photochem. Photobiol. B Biol..

[41] Cobb500 Broiler Performance & Nutrition Supplement. https://www.cobb-vantress.com/en_US/products/cobb500/.

[42] AOAC (Association of Official Analysis Chemists International) (2005). Official Methods of Analysis of AOAC International.

[43] Romero L.M., Reed J.M. (2005). Collecting baseline corticosterone samples in the field: Is under 3 min good enough?. Comp. Biochem. Physiol.-A Mol. Integr. Physiol..

[44] Zhang L., Yue H.Y., Zhang H.J., Xu L., Wu S.G., Yan H.J., Gong Y.S., Qi G.H. (2009). Transport stress in broilers: I. Blood metabolism, glycolytic potential, and meat quality. Poult. Sci..

[45] Gehad A.E., Mehaisen G.M.K., Abbas A.O., Mashaly M.M. (2008). The Role of Light Program and Melatonin on Alleviation of Inflammation Induced by Lipopolysaccharide Injection in Broiler Chickens. Int. J. Poult. Sci..

[46] Alaqil A.A., Abbas A.O., El-Beltagi H.S., Abd El-Atty H.K., Mehaisen G.M.K., Moustafa E.S. (2020). Dietary supplementation of probiotic lactobacillus acidophilus modulates cholesterol levels, immune response, and productive performance of laying hens. Animals.

[47] Loa C.C., Lin T.L., Wu C.C., Bryan T., Thacker H.L., Hooper T., Schrader D. (2001). Humoral and cellular immune responses in turkey poults infected with turkey coronavirus. Poult. Sci..

[48] Jahanian R., Rasouli E. (2015). Dietary chromium methionine supplementation could alleviate immunosuppressive effects of heat stress in broiler chicks. J. Anim. Sci..

[49] Akhavan-Salamat H., Ghasemi H.A. (2016). Alleviation of chronic heat stress in broilers by dietary supplementation of betaine and turmeric rhizome powder: Dynamics of performance, leukocyte profile, humoral immunity, and antioxidant status. Trop. Anim. Health Prod..

[50] Habibian M., Ghazi S., Moeini M.M., Abdolmohammadi A. (2014). Effects of dietary selenium and vitamin E on immune response and biological blood parameters of broilers reared under thermoneutral or heat stress conditions. Int. J. Biometeorol..

[51] Sohail M.U., Ijaz A., Yousaf M.S., Ashraf K., Zaneb H., Aleem M., Rehman H. (2010). Alleviation of cyclic heat stress in broilers by dietary supplementation of mannan-oligosaccharide and Lactobacillus-based probiotic: Dynamics of cortisol, thyroid hormones, cholesterol, C-reactive protein, and humoral immunity. Poult. Sci..

[52] Charmandari E., Tsigos C., Chrousos G. (2005). Endocrinology of the stress response. Annu. Rev. Physiol..

[53] Villar S.R., Ronco M.T., Fernández Bussy R., Roggero E., Lepletier A., Manarin R., Savino W., Pérez A.R., Bottasso O. (2013). Tumor Necrosis Factor-α Regulates Glucocorticoid Synthesis in the Adrenal Glands of Trypanosoma cruzi Acutely-Infected Mice. The Role of TNF-R1. PLoS ONE.

[54] Li G.M., Liu L.P., Yin B., Liu Y.Y., Dong W.W., Gong S., Zhang J., Tan J.H. (2020). Heat stress decreases egg production of laying hens by inducing apoptosis of follicular cells via activating the FasL/Fas and TNF-α systems. Poult. Sci..

[55] Webster Marketon J.I., Glaser R. (2008). Stress hormones and immune function. Cell. Immunol..

[56] Belhadj Slimen I., Najar T., Ghram A., Abdrrabba M. (2016). Heat stress effects on livestock: Molecular, cellular and metabolic aspects, a review. J. Anim. Physiol. Anim. Nutr..

[57] De Oliveira R.G., Lara L.J.C. (2016). Lighting programmes and its implications for broiler chickens. Worlds. Poult. Sci. J..

[58] Rodriguez C., Mayo J.C., Sainz R.M., Antolín I., Herrera F., Martín V., Reiter R.J. (2004). Regulation of antioxidant enzymes: A significant role for melatonin. J. Pineal Res..

[59] Li J.H., Yu J.P., Yu H.G., Xu X.M., Yu L.L., Liu J., Luo H.S. (2005). Melatonin Reduces Inflammatory Injury Through Inhibiting NF-κB Activation in Rats With Colitis. Mediat. Inflamm..

[60] Leal M., Shimada A., Ruíz F., De Mejía E.G. (1999). Effect of lycopene on lipid peroxidation and glutathione-dependent enzymes induced by T-2 toxin in vivo. Toxicol. Lett..

[61] Alzarah M.I., Alaqil A.A., Abbas A.O., Nassar F.S., Mehaisen G.M.K., Gouda G.F., Abd El-Atty H.K., Moustafa E.S. (2021). Inclusion of citrullus colocynthis seed extract into diets induced a hypolipidemic effect and improved layer performance. Agriculture.

[62] Mauss D., Herr R.M., Jarczok M.N., Motoc I., Fischer J.E., Bosch J.A. (2021). The association of cortisol levels with leukocyte distribution is disrupted in the metabolic syndrome. Obes. Res. Clin. Pract..

[63] Shini S., Huff G.R., Shini A., Kaiser P. (2010). Understanding stress-induced immunosuppression: Exploration of cytokine and chemokine gene profiles in chicken peripheral leukocytes. Poult. Sci..

[64] Carrillo-Vico A., Lardone P.J., Álvarez-Śnchez N., Rodrĩguez-Rodrĩguez A., Guerrero J.M. (2013). Melatonin: Buffering the immune system. Int. J. Mol. Sci..

[65] Charlton M.R. (1996). Protein metabolism and liver disease. Baillieres. Clin. Endocrinol. Metab..

[66] Siegel H.S., Van Kampen M. (1984). Energy relationships in growing chickens given daily injections of corticosterone. Br. Poult. Sci..

[67] Mathivanan R., Edwin S.C. (2012). Hematological and serum biochemical parameters of broilers fed with Andrographis paniculata as an alternative to antibiotic growth promoter. J. Med. Plants Res..

[68] Darras V.M., Kotanen S.P., Geris K.L., Berghman L.R., Kühn E.R. (1996). Plasma thyroid hormone levels and iodothyronine deiodinase activity following an acute glucocorticoid challenge in embryonic compared with posthatch chickens. Gen. Comp. Endocrinol..

[69] Mustonen A.M., Nieminen P., Hyvarinen H., Asikainen J. (2000). Exogenous melatonin elevates the plasma leptin and thyroxine concentrations of the mink (*Mustela vison*). Z. Naturforsch. C.

[70] Schwean-Lardner K., Fancher B.I., Classen H.L. (2012). Impact of daylength on the productivity of two commercial broiler strains. Br. Poult. Sci..

