# Supplementary material for: Intermittent Lighting Program Relieves the Deleterious Effect of Heat Stress on Growth, Stress Biomarkers, Physiological Status, and Immune Response of Broiler Chickens
Source: Animals (Basel). 2022 Jul 19;12(14):1834. doi: 10.3390/ani12141834 (PMC9311685; doi:10.3390/ani12141834)
Supplement: Supplementary file 1 [file animals-12-01834-s001.zip › animals-1659794-supplementary.pdf]

File S1

Stress biomarkers assay:

Corticosterone (CORT)

Plasma CORT was assayed according to the manufacturer protocol for a chicken ELISA kit (MBS701668, MyBioSource, Inc., San Diego, CA, USA. The principal of the CORT assay is based on mixing 50 μL of the standard or the sample with 50 μL of the antibody in pre-coated antigen microplate wells. Following a 30-minute incubation period at 25°C, the wells were aspirated and washed three times with 250 μL of washing buffer, with the residues removed by flipping the plate and blotting it on clean paper towels. Each well received 100 μL of horseradish peroxidase (H.R.P.) conjugated reagent, which was heated to around 25°C for 30 min. A quantity of 100 μL T.M.B. ELISA-substrate was added to each well in the dark and incubated for another 15 minutes. Subsequently, 50 μL of the stop-reaction solution was added to each well and gently mixed, and the optical density was measured using a microplate reader at 450 nm (ELx808™ BioTek Instruments, Winooski, VT, USA). CORT analysis sensitivity and detection ranges were <0.5 and 0.5-20.0 ng/ml, respectively. The intra- and inter-assay CV% were <8% and <10%, respectively

Tumor necrosis factor alpha (TNF-α)

Plasma TNF-α was measured according to the manufacturer's procedure for a chicken ELISA kit (MBS2509660, MyBioSource, Inc.). Briefly, 100 μL of the standard or sample was put into each well of the pre-coated antigen microplate and incubated at 37°C for 90 minutes following the removal of the liquid. The wells were filled with 100 μL of biotinylated detection antibody and incubated at 37°C for 1 hour. After washing three times, 100 μL of H.R.P. conjugate was added to the plate, then incubated for another 30 minutes before being aspirated and washed five times more. The substrate reagent was added to the wells in 90 μL increments, incubated for 15 minutes at 37°C, and then the stop solution was added in 50 μL increments to each well. Lastly, the optical density was measured soon after the last step at 450 nm using the microplate reader. The TNF-α assay has sensitivity and detection ranges of 18.75 and 31.25-2000.00 pg/ml, respectively, and intra- and inter-assay CV percentages of 5.57% and 5.89%, respectively, and the intra- and inter-assay CV% were <5.57% and <5.89%, respectively.

Malondialdehyde (MDA)

A colorimetric test kit was used to determine the lipid peroxidation product (MDA) in the plasma (MBS9718963, MyBioSource, Inc., San Diego, CA, USA). A quantity of 100 μL plasma was combined with 300 μL of thiobarbituric acid (T.B.A.) solution and incubated for 30 minutes at 95 degrees Celsius, according to the manufacturer's instructions. The mixture was centrifuged at 10000 x g for 10 minutes at 25 °C after chilling in an ice bath for 10 minutes. The absorbance was measured at 532 nm using a microplate reader after aliquots of 200 μL of the supernatants were transferred to a 96-well microplate.

Blood metabolites assay:

Total protein (TP)

The Bradford procedure assay kit detected plasma TP (ab102535, Abcam, Waltham, MA, USA). In a clear-bottom 96-well plate, 100 μL of the working solution (1X) was added to duplicate either 10 μL of diluted standards or 10μL of diluted samples. The materials were gently mixed before being incubated at room temperature for 5 minutes. This reaction produced a blue complex detected at 595 nm, and the sample protein content was calculated using the standard curve.

Aspartate aminotransferase (AST) activity

Plasma AST levels were determined using available colorimetric kits (ab241035, Abcam). For the AST assay, up to 50 μL of the diluted samples or glutamate standards were mixed well with 100 μL of a reaction mix (80 μL AST buffer, 2 μL enzyme mix, 8 μL developer, and 10 μL AST substrate) into a 96-well plate. The optical density at 450 nm was obtained after 10 min and again after 60 min incubation at 37°C. The AST activity was determined by calculating the amount of glutamate generated by AST per min.

Alanine aminotransferase (ALT) activity

Plasma ALT levels were determined using available colorimetric kits (ab105135, Abcam). For the ALT assay, up to 5 μL of the diluted samples or pyruvate standards were mixed well with 25 μL of a reaction mix (21.5 μL ALT buffer, 0.5 μL enzyme mix, 0.5 μL red prob, and 2.5 μL ALT substrate) into a 96-well plate. The optical density at 570 nm was obtained after 10 min and again after 60 min incubation at 37°C. The ALT activity was determined by calculating the amount of pyruvate generated by ALT per min.

Chicken triiodothyronine (chT$_3$)

The concentration of T$_3$ in the plasma was measured according to the guidelines of the ELISA kit's specifications for chickens (MBS269454; MyBioSource, Inc.). The kit's reagents were first balanced to room temperature for at least 20 min before conducting the test. One hundred μL of the samples or chT$_3$ standards were added to the microplate wells and incubated for 90 minutes at 37°C. After two washes, 100 μ L of biotinylated chT3 antibody was added to each well and incubated for 60 minutes at 37°C. After three washes, 100 μL of enzyme-conjugate was applied to every well, and it was heated at 37°C for 30 minutes. After washing 5 times, 100 mL of color reagents A and B (9:1 v/v) were added to each well and incubated in the dark at 37°C until the standard solution color gradients developed. Finally, 100 μL of color reagent C was added to each well, mixed thoroughly, and the optical density at 450 nm was measured after 10 minutes. For this analysis, the intra- and inter-assay CV% were <8% and 12%, respectively, with a sensitivity of 0.06 μmol/mL and a detection range of 0.3–20 μmol/mL.

Immune response parameters assay:

Total white blood cells (TWBC)

At the end of the 42-day experiment, blood samples were taken from broiler birds (n=10; 2 samples were taken from each replicate in each treatment group) and put into heparinized tubes. A drop of the entire sample (10 µL) was diluted with brilliant cresyl blue stain solution (1:50 v/v). A mixture drop was put on a Bright-Line™ hemocytometer slide (American Optical, Buffalo, NY, USA). A microscope with a magnification of 200X was used to look at the TWBC and count them (Gehad et al., 2008).

Lymphocyte proliferation index

The remaining blood samples (n=10) were used to measure the stimulation index (SI) of T- and B-lymphocyte proliferation, as reported in a recent study (Alaqil et al., 2020). First, the peripheral blood mononuclear cells (PBMC) were separated by overlaying the blood samples on the top of an equivalent volume of a separation medium (Histopaque-1077, Sigma Chemical Co., St. Louis, MO, USA) in a falcon tube and centrifuging at 1030 x g for 20 min at 4°C. The isolated layers of the PBMC were carefully aspirated from the interface of the opaque medium and with RPMI-1640 medium washed twice (Invitrogen Corp., Grand Island, NY, USA), and then resuspended in 1 mL of RPMI-1640. The concentration of viable lymphocytes in each sample was detected by Trypan Blue dye and then re-adjusted at 10 million cells/mL in triplicates in 96-well plates. To promote T- or B-lymphocyte proliferation, experimental wells were supplied with 50 µL of either 5% Concanavalin-A mitogen or 1% Lipopolysaccharide, whereas control wells were treated with 50 µ L RPMI-1640. After 48 hours of incubation at 42°C, 5% CO2, and saturated humidity, 15 µL of 3-[4,5-dimethylthiazol]-2,5-diphenyltetrazolium bromide (MTT 0.5 percent, Sigma) was added to each well and incubated for another 4 hours. Finally, each well was filled with 100 µL of 10% sodium dodecyl sulfates dissolved in 0.04 M HCl, and the optical density at 570 nm (OD570) was measured for the experimental and control wells using a microplate reader. The ratio of OD570 for stimulated to unstimulated cells in each sample was used to compute the SI of T- and B-lymphocytes.

Antibody titers against sheep red blood cells (Anti-SRBCs AB)

Broiler Anti-SRBCs AB titers were determined using procedures outlined in a prior study (Loa et al., 2001). In short, one week before the end of the experiment, two birds per replication in each treatment group (n=10) were injected with 1 mL of 5 percent SRBC. Blood samples were taken from the birds at 42 days of age, and the sera were separated by centrifugation at 220 x g at room temperature. In a 96-well plate, serial doubling dilutions of sera samples (25 µL each) were pipetted, with 25 µL of 2% SRBC solution added to each dilution. For a few seconds, the plates were gently shaken. They were then left at room temperature overnight to make them clump together. The AB titer of Ant-SRBCs was calculated as $\log_2$ of the inverse of the last dilution with positive agglutination in the well's bottom.

References

Alaqil, A. A., A. O. Abbas, H. S. El-Beltagi, H. K. Abd El-Atty, G. M. K. Mehaisen, and E. S. Moustafa. 2020. Dietary supplementation of probiotic lactobacillus acidophilus modulates cholesterol levels, immune response, and productive performance of laying hens. Animals 10:1–12 Available at https://pubmed.ncbi.nlm.nih.gov/32899938/ (verified 5 April 2021).

Gehad, A. E., G. M. K. Mehaisen, A. O. Abbas, and M. M. Mashaly. 2008. The Role of Light Program and

Melatonin on Alleviation of Inflammation Induced by Lipopolysaccharide Injection in Broiler Chickens. Int. J. Poult. Sci. 7:193–201 Available at http://www.scialert.net/abstract/?doi=ijps.2008.193.201.

Loa, C. C., T. L. Lin, C. C. Wu, T. Bryan, H. L. Thacker, T. Hooper, and D. Schrader. 2001. Humoral and cellular immune responses in turkey poults infected with turkey coronavirus. Poult. Sci. 80:1416–1424 Available at http://www.ncbi.nlm.nih.gov/pubmed/11599699.
